# Evaluation of a novel portable capacitive ECG system in the clinical practice for a fast and simple ECG assessment in patients presenting with chest pain: FIDET (Fast Infarction Diagnosis ECG Trial)

**DOI:** 10.1007/s00392-012-0512-7

**Published:** 2012-09-29

**Authors:** Eva C. L. Rasenack, Martin Oehler, Albrecht Elsässer, Meinhard Schilling, Lars S. Maier

**Affiliations:** 1 Department of Cardiology and Pneumology/Heart Center, Georg-August-University Göttingen, Robert-Koch-Str. 40, 37075 Göttingen, Germany; 2 Capical GmbH, Braunschweig, Germany; 3 Clinic for Cardiology/Heart Center Oldenburg, Oldenburg, Germany; 4 Institute of Electrical Measurement and Fundamental Electrical Engineering, TU, Braunschweig, Germany

**Keywords:** Capacitive ECG system, Capacitive electrodes, Myocardial infarction, Acute coronary syndrome, Clinical cardiology, ECG assessment

## Abstract

**Background:**

Electrocardiogram (ECG) assessment plays a crucial role in patients presenting with chest pain and suspected acute coronary syndrome (ACS). In a pilot study, we previously evaluated a capacitive ECG system (cECG) as a novel ECG technique for a fast and simple ECG assessment in patients with ST-elevation myocardial infarction (STEMI). In a next step, the sensitivity and specificity of this novel ECG technique have to be assessed in patients with ACS.

**Hypothesis:**

The Fast Infarction Diagnosis ECG Trial (FIDET) is a prospective, bi-center, observer-blinded noninferiority study to evaluate the cECG compared to the conventional ECG (kECG) in the clinical practice for ECG assessment in consecutive patients presenting with suspected ACS.

**Methods:**

In 250 patients who were admitted to the hospital, because of an ACS [including STEMI and non-ST-elevation acute coronary syndrome (NSTE-ACS)], both a kECG and a cECG recording were performed within a time lag of less than 10 min.

**End points:**

The primary end point will be sensitivity and specificity of the cECG compared to the kECG in diagnosing a STEMI with a margin of noninferiority of 7.5 %. Secondary end points include sensitivity and specificity of the cECG compared to the kECG in diagnosing an NSTE-ACS, safety of the cECG system (adverse event, serious adverse event and suspected unexpected serious adverse reaction), parameters of the ECG measurement (PQ-interval, QT-interval, ST-amplitude and heart rate) and measurement duration of the two methods.

**Conclusion:**

FIDET is designed as a noninferiority study to show that a novel cECG system is suitable for the diagnosis of myocardial infarction in the clinical context and might even have benefits, for example by offering a faster and easier ECG assessment.

## Introduction

In 1902, Willem Einthoven published his first recordings of the heart from his “string galvanometer” which he named “elektrocardiogramm” [1]. In the past century, the electrocardiogram (ECG) became one of the most frequently used diagnostic devices all over the world and plays a crucial role in the diagnosis of acute myocardial infarction (MI) [2, 3]. This is especially important in the pre-hospital phase, where an early diagnosis of an ST-elevation myocardial infarction (STEMI) sets the course for the rapid initiation of reperfusion therapy [4].

A so-called capacitive ECG system (cECG) is a novel technical innovation, which could be a useful diagnostic tool for a fast and simple ECG assessment. Unlike the conventional 12-lead ECG (kECG), there is no need to fix the electrodes to the skin and no need of electrolyte gel. This might be very useful in an emergency situation when a fast diagnosis is especially important, e.g., in patients presenting with an acute coronary syndrome (ACS). In hemodynamically unstable patients admitted to the hospital with shock or pre-shock, the adhesion of the conventional electrodes to the wet skin sometimes is hardly possible. In this situation, the application of a cECG system could be beneficial as well.

In a proof of concept pilot study that was performed between March 2009 and May 2010, we could show that it was possible to identify a STEMI with the cECG in clinical practice [6]. In 66 patients admitted to the hospital, because of a STEMI a cECG was performed and compared to a kECG of the same patient. During the course of the study, the cECG was modified, because the initially used ECG system was able to detect an anterior MI without a problem, but did not identify an inferior MI sufficiently. After adding an additional electrode further away from the electrodes on the chest at the lower back of the patient, ST-elevations in the inferior leads II, III and aVF could be assessed appropriately as well. After these first promising clinical results, further studies are needed to analyze the sensitivity and specificity of the novel innovative ECG technique.

## Methods

### Study design

The FIDET study is a prospective, bi-center, observer-blinded, single-arm noninferiority study, where the participant is the control group at the same time (protocol ID 20110819-MO, EUDAMED-No. CIV-11-08-002121). Our intention is to evaluate the cECG compared to the kECG in the clinical practice for ECG assessment in consecutive patients presenting with ACS with regard to reliability and speed. The trial was funded by Capical GmbH, Braunschweig, Germany. It was approved by the ethics committee of the University Medical Center Göttingen as the leading ethics committee. As the measurements were carried out in patients who were in a potentially unstable state, where no time should be wasted, we got permission to obtain written informed consent after the ECG assessments.

### Conducting patient selection and study

**Fig. 1:**
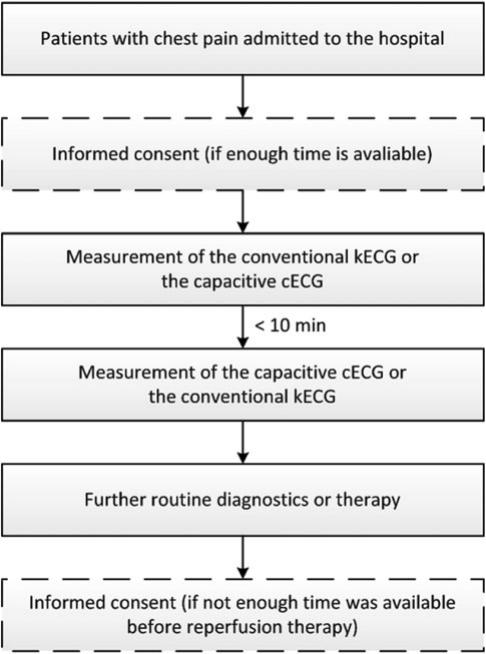
Flowchart of the study protocol

The duration of the study is supposed to be very short. Consecutive patients presenting to the hospital with ACS will be screened for inclusion into the study. Taking the different entities of ACS together (STEMI, NSTE-ACS), measurements will presumably be conducted in the emergency room (ER), the cardiac catheterization laboratory (before reperfusion therapy is started) and the chest pain unit (CPU). Inclusion and exclusion criteria are listed in Table 1. If a patient fulfills the inclusion and exclusion criteria, both a cECG and a kECG will be performed with a time lag of less than 10 min (flowchart of the protocol, Fig. 1). We choose this short interval to make the different ECG assessments as comparable as possible. There will be neither any further examinations nor a follow-up, but some of the patients’ personal data will be collected in the electronic case report forms (eCRF) for further analysis. We intend to include 250 patients. An interim safety analysis will be performed after recruitment of the first 100 patients.

**Table 1:** Inclusion and exclusion criteria

Inclusion criteria	Exclusion criteria
Male and female patients ≥18 years	Pregnant or breastfeeding women
Patients presenting with chest pain and suspected ACS (acute coronary syndrome)	Patients carrying a pacemaker (PM) or implantable cardioverter defibrillator (ICD)
Signed written informed consent	Bundle-branch block visible in the ECG (except for a new left bundle-branch block)
A 12-lead ECG is routinely recorded in the hospital on admission of the patient	Cardiogenic shock
	Patients who recently underwent an operation at the chest

### Medical device (capacitive ECG system) and measuring procedure

**Fig. 2:**
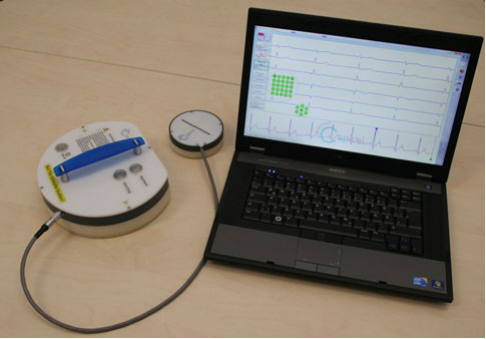
The cECG device used consists of a sensor array with 25 flexible mounted capacitive electrodes integrated into a ~20 × 20 cm round mainframe and another 7 electrodes on the additional probe connected via cable to the mainframe. The recording is automatically sent to the laptop via Bluetooth connection

The used cECG system is shown in Fig. 2; it consists of a sensor array with 25 flexible mounted capacitive electrodes integrated into a ~20 × 20 cm round mainframe and another 7 electrodes on the additional probe connected via cable to the mainframe.

While the measuring principle of the cECG and the kECG is the same, both detect potential differences during the electric heart action and the particular sensor (capacitive or galvanic electrode) works in a different mode. Capacitive electrodes, among other things for ECG measurements, were first described in the 1960s [7–9]. In contrast to the conventional galvanic electrodes that require an electrolyte gel for enabling a certain conductivity for measuring a bioelectric signal, they are isolated from the body and provide a capacitive coupling between the skin and a metallic face inside the sensor. The signal is injected in a capacitive way with high impedance, as the electrode detects electric displacement currents that are caused by the changing potential distribution during the heartbeat. For this purpose, a special amplifier has to be connected to the metallic face.

With this configuration, it is possible to obtain an ECG recording with the cECG that gives exactly the same information as the kECG when the electrodes are placed at the same position, and comparable results if the capacitive electrodes are placed in an array like the cECG system used in this and previous studies [5, 10]. After modification of the cECG system in the course of our previous pilot study [6], the current clinical trial will be conducted with model number 2.0 and the software version 1.0.

**Fig. 3:**
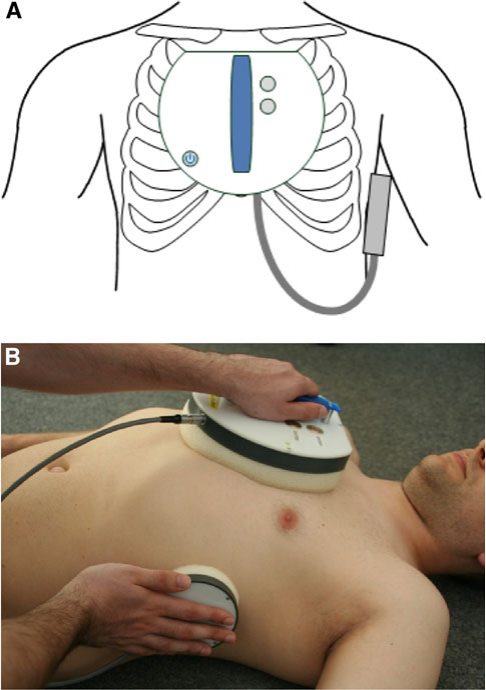
Correct position of the cECG. **a** Schematic position of the cECG device on the chest and **b** demonstration of an ECG assessment

For ECG assessment, the system is placed on the patient’s undressed chest, the mainframe central on the chest in the range of the third intercostal space and the additional probe on the left side of the patient in the range of the fifth intercostal space in the midaxillary line (MAL) with the upper electrode on the Wilson V6 position (Fig. 3). The ECG recording is then controlled and saved by the mainframe, allowing a fast and simple handling. Once a recording is saved, it is automatically sent to the laptop via Bluetooth connection, where a further processing of the data is possible.

### End points

The FIDET study will include the following end points:

1. Primary end point: sensitivity and specificity of the cECG in the detection of a STEMI compared to the international gold standard (kECG)
2. Secondary end points
  - sensitivity and specificity of the cECG in diagnosing an NSTEMI compared to the gold standard (kECG, biomarker)
  - safety of the cECG system; documented by adverse events (AE), serious adverse events (SAE), occurrences, undesirable effects and serious undesirable effects of the product
  - different parameters of the ECG measurement (PQ-interval, QT-interval, ST-amplitude and heart rate)
  - measurement duration of the two different methods (kECG vs. cECG)
  - diagnostic information of space-resolved ECG data of all channels of the cECG via temporal and local analysis in comparison to the kECG as gold standard

### Analysis and statistical considerations

The collected data will be acquired in an electronic CRF. The study database will be created by using the software secuTrail^©^. As mentioned above, the FIDET study is designed as a prospective, bi-center, observer-blinded, single-arm noninferiority study to prove the suitability of the cECG for the diagnosis of MI in the clinical context. The cECG will be compared directly with the 12-lead ECG as the current gold standard for the diagnosis of MI. For analysis, evaluation of a 12-lead ECG by an investigator of one center will be compared to the cECG of the same patient evaluated by an investigator of the other study site. As the study is observer blinded, the evaluation of the cECG will be carried out without knowing the result of the kECG; therefore, there is no need for a control group.

The sensitivity and specificity of the cECG in detecting a STEMI compared to the gold standard (kECG) will be determined using a fourfold table and 95 % confidence intervals will be calculated. A *p* value of <0.05 will be considered as statistically significant. The sensitivity of the cECG in STEMI detection was estimated to be 92.5 % based on the data from the prior study. Therefore, 84 patients with STEMI are needed to stay within the 95 % confidence interval, choosing a 7.5 % tolerance level. Corresponding results hold true for specificity. Based on an expected STEMI prevalence of 35 % in the patient population, a sample size for this study of approximately 250 patients should be adequate to sufficiently match the requirements for the confidence interval calculation regarding sensitivity and specificity.

## Discussion

One of the most common emergency situations is a patient presenting with chest pain. Unless proven otherwise, an ACS is suspected that requires a rapid diagnosis to initiate further therapy due to the impaired prognosis for patients with a possible MI. The 12-lead ECG is the most important diagnostic tool in this setting to distinguish between the different diseases involving the coronary arteries STEMI, NSTEMI or unstable angina (the latter two are newly called NSTE-ACS) [11]. In case of STEMI, a reperfusion therapy is indicated as soon as possible [4, 12].

Thus, new ECG techniques that offer a faster and easier ECG assessment are developed [13]. A portable, so-called cECG system based on capacitive electrodes permits a measurement of ECG signals without fixing the electrodes on the patient’s skin or using electrolyte gel. To show that the cECG is a promising diagnostic tool in the clinical environment, we performed a pilot study in 66 patients admitted to the hospital with a STEMI. After modification of the cECG, we were able to detect both anterior and inferior MI. To analyze the sensitivity and specificity and further optimize the cECG system, the FIDET study is planned as a noninferiority study to prove that the innovative ECG technique is suitable to diagnose MI in the clinical context. A noninferiority margin of 7.5 % was allowed. Therefore, a kECG as well as a cECG will be performed in consecutive patients admitted to the hospital with chest pain, thereby allowing a direct comparison between the two types of ECG examinations. A total of 250 patients will be included. The primary end point of the study will be sensitivity and specificity of the cECG compared to the kECG in diagnosing a STEMI. The primary end point is defined as sensitivity and specificity for STEMI diagnosis of the capacitive ECG compared to the gold standard 12-channel ECG. Per definition, the sensitivity and specificity of the 12-channel ECG is 100 %. Further, we will analyze the results of the coronary angiography of the STEMI patients to validate the sensitivity of the 12-channel ECG. This is not possible for specificity, because not all 250 patients underwent a coronary angiography. In a third step, the 12-channel ECG and the capacitive ECG will be compared to enzyme elevations. Basically, the treatment for the individual patient is very short and simple and a follow-up is not planned.

If the cECG can demonstrate its ability to reliably diagnose an MI, it might be a quite useful diagnostic device in hospital and especially in the pre-hospital phase, where a rapid diagnosis is required [14]. An integration of the cECG system into the patient monitor used in the emergency medical service, for example, would make the assessment of ECG and the subsequent transmission to a PCI center by the cardiologist on duty very fast, easy and straightforward and may even further improve outcome [15].

In conclusion, a rapid diagnosis in patients presenting with chest pain is of utmost importance. The so-called capacitive ECG system, a novel ECG technique with capacitive electrodes, was able to identify STEMIs in a pilot study. The FIDET was designed as a noninferiority study to investigate sensitivity and specificity of cECG and thus prove that it is suitable for diagnosing MI in clinical practice and might even have benefits by offering a particularly fast and simple ECG assessment.

## Abbreviations

ACS: Acute coronary syndrome
AE: Adverse event
cECG: Capacitive ECG system
CPU: Chest pain unit
eCRF: Electronic case report form
ECG: Electrocardiogram
ER: Emergency room
ICD: Implantable cardioverter defibrillator
kECG: Conventional (12-lead) ECG
MAL: Midaxillary line
MI: Myocardial infarction
NSTE-ACS: Non-ST-elevation acute coronary syndrome
NSTEMI: Non-ST-elevation myocardial infarction
PM: Pacemaker
SAE: Serious adverse event
STEMI: ST-elevation myocardial infarction
SUSAR: Suspected unexpected serious adverse reaction
